# The Baby Bridge program: A sustainable program that can improve therapy service delivery for preterm infants following NICU discharge

**DOI:** 10.1371/journal.pone.0233411

**Published:** 2020-05-29

**Authors:** Roberta Pineda, Elizabeth Heiny, Patricia Nellis, Joan Smith, Jaqueline M. McGrath, Margaux Collins, Abigail Barker

**Affiliations:** 1 Chan Division of Occupational Science and Occupational Therapy, University of Southern California, Los Angeles, California, United States of America; 2 Department of Pediatrics, Keck School of Medicine, University of Southern California, Los Angeles, California, United States of America; 3 Program in Occupational Therapy, Washington University School of Medicine, St. Louis, Missouri, United States of America; 4 Department of Safety, Quality, and Practice Excellence, St. Louis Children’s Hospital, St Louis, Missouri, United States of America; 5 School of Nursing, University of Texas Health Science Center, San Antonio, Texas, United States of America; 6 Center for Health Economics and Policy, Institute for Public Health, Brown School, Washington University, St Louis, Missouri, United States of America; Murdoch University, AUSTRALIA

## Abstract

**Objective:**

The aim of this project was to determine revenues and costs over time to assess the sustainability of the Baby Bridge program.

**Methods:**

The Baby Bridge program was developed to promote timely, consistent and high quality early therapy services for high-risk infants following neonatal intensive care unit (NICU) discharge. Key features of the Baby Bridge program were defined as: 1) having the therapist establish rapport with the family while in the NICU, 2) scheduling the first home visit within one week of discharge and continuing weekly visits until other services commence, 3) conducting comprehensive assessments to inform targeted interventions by a skilled, single provider, and 4) using a comprehensive therapeutic approach while collaborating with the NICU medical team and community therapy providers. The Baby Bridge program was implemented with infants hospitalized in an urban Level IV NICU from January 2016 to January 2018. The number of infants enrolled increased gradually over the first several months to reach the case-load capacity associated with one full-time therapist by mid-2017. Costs of the therapists delivering Baby Bridge services, travel, and equipment were tracked and compared with claim records of participants. The operational cost of Baby Bridge programming at capacity was estimated based on the completed and anticipated claims and reimbursement of therapy services as a means to inform possible scale-ups of the program.

**Results:**

In 2016, the first year of programming, the Baby Bridge program experienced a loss of $26,460, with revenue to the program totaling $11,138 and expenses totaling $37,598. In 2017, the Baby Bridge program experienced a net positive income of $2,969, with revenues to the program totaling $53,989 and expenses totaling $51,020. By Spring 2017, 16 months after initiating Baby Bridge programming, program revenue began to exceed cost. It is projected that cumulative revenue would have exceeded cumulative costs by January 2019, 3 years following implementation. Net annual program income, once scaled up to capacity, would be approximately $16,308.

**Discussion:**

There were initial losses during phase-in of Baby Bridge programming associated with operating far below capacity, yet the program achieved sustainability within 16 months of implementation. These costs related to implementation do not consider the potential cost reduction due to mitigated health burden for the community and families, particularly due to earlier receipt of therapy services, which is an important area for further inquiry.

## Introduction

Preterm infants have a high risk of developmental delays, including motor, cognitive, and language difficulties, as well as behavioral and learning problems [1–3]. The Center for Disease Control (CDC) documented that preterm birth is the leading cause of long-term disability, as well as a significant source of emotional and economic burden for families [4]. Potential neurodevelopmental impairment is already present at the time of discharge from the neonatal intensive care unit (NICU) for many high-risk infants [5–9]. Infants with identified neurodevelopmental impairment are at risk of long-term disability; therefore, they often receive a referral for physical therapy (PT), occupational therapy (OT), and/or speech-language pathology (SLP) prior to NICU discharge [10, 11]. Early intervention and therapy programs are beneficial for improving outcomes in children who are born preterm and have alterations in neurodevelopment [12], and early therapy services are believed to be most beneficial [13]. When sensory and motor experiences are impaired due to an adverse environment or alterations in development, different patterns of experiences emerge that can impact early learning and skill acquisition [14]. Timing of specific sensory and motor exposures that align with normal developmental patterns is critical during the first few months of life [14], as they drive the emergence of an abundance of positive, developmentally advantageous synapses, laying the foundation for later pruning for specialization. Early therapy can drive appropriate neuronal activity during a critical period of development.

Although therapy referrals at NICU discharge are the standard of care in most NICU settings [15–17] and despite evidence supporting early therapy interventions and policy-mandated provision of services to at-risk infants [18], therapy is often difficult to access [19–21]. Preterm infants with neurodevelopmental impairment referred for therapy at the time of NICU discharge may wait an average of four to five months before they receive services [22]. Even when referrals are made prior to discharge from the NICU, other socioeconomic barriers to early therapy services exist, including low income, low maternal education, and single-family households [23]. Further, Feinberg et al has identified that Black children are five times less likely to access early intervention services than White children [24]. From a payer perspective, evidence on access to early therapy for children with Medicaid versus private insurance is mixed [19, 23, 25, 26]. Low referral rates, lack of family follow-through, and stigma associated with utilizing services can result in under-enrollment of services for extremely low birth weight children, especially among minorities and those with high social risk [27]. However, infants with high social risk have an even higher likelihood of developmental challenges [28–32], and implementing programming to improve access and optimize function can have far-reaching effects.

The Baby Bridge program was developed as an implementation strategy aimed at ensuring early and continuous therapy services following NICU discharge for preterm infants with alterations in neurodevelopment until other community-based therapy services commence. The Baby Bridge program utilizes a specialized licensed therapist who sees the infant and family in the NICU prior to discharge, completes a comprehensive neurodevelopmental assessment to inform targeted interventions, and provides early therapy services in the home environment within one week of discharge and weekly thereafter, until other community-based services are initiated. The therapist also educates the family on ways to support their infant’s neurodevelopment between sessions and provides support and assistance during the transition from hospital to home. The Baby Bridge therapist also uses a comprehensive therapeutic approach and collaborates with the NICU medical team and community therapy providers.

Previous work has demonstrated improvements in access to early therapy services with Baby Bridge programming [33]. This previous work demonstrated that Baby Bridge programming was an effective implementation strategy and was feasible to implement. The Baby Bridge program resulted in more infants receiving therapy services after NICU discharge (n = 58/60; 97% compared to n = 44/57; 77%; p <0.0001). Infants in the Baby Bridge program received therapy an average of 85 days earlier [<0.0001; β = -84.7 (-70.2 to -99.2)] than controls, demonstrating that when Baby Bridge programming is used as an implementation strategy, it can improve access to care. However, cost concerns are negatively associated with successful implementation [34], and according to the Consolidated Framework for Implementation Research (CFIR), cost is an important construct to measure and report.

In healthcare, many programs are implemented because they are believed to be beneficial, even when they are not cost effective. However, many programs fail to be implemented or are discontinued due to concerns about cost or to lack of return on investment. In order for new programming to become a standard of care, cost is an issue, especially during a time when health care organizations are seeking to reduce costs. Understanding the cost versus benefit is important for others who may consider implementing Baby Bridge or similar programming.

This study aims to calculate the costs of the Baby Bridge program to compare it against revenues in order to assess sustainability for possible scale-up across sites.

## Methods

This study was approved by the study site institutional review board.

This study compared the total costs and revenues of Baby Bridge programming over a span of 2 years. Infants were enrolled in the Baby Bridge program and received weekly therapy services in their homes until other community-based (early intervention) services commenced. Therapy services were billed within a therapy practice using standard billing paradigms. Total cost of the therapist, mileage and travel expenses, and equipment were calculated. Claims were then compared to cost over time to assess sustainability of Baby Bridge programming.

### Participants

This study enrolled 95 high-risk infants who were being discharged from the NICU at St. Louis Children’s Hospital. Infants were recruited prior to discharge home. Infants were excluded for the following reasons: therapy was not recommended by the medical team at discharge, the infant did not receive therapy in the NICU, the family resided in a county that was greater than 50 miles away from St. Louis Children’s Hospital, the family lived outside of Missouri, the infant was a ward of the state, or the family did not speak English. All infants who were eligible for the study also qualified for early intervention services through the Individuals with Disabilities Act (IDEA) Part C. These are therapy services provided for children from birth to 3 years old who are at risk of developmental disabilities. For the first 6 months of Baby Bridge programming, inclusion criteria consisted of preterm infants born between 28–30 weeks estimated gestational age. After the first 6 months, as program capacity increased, the criteria were expanded to include preterm infants born ≤30 weeks gestation. In the final year of implementation, the inclusion criteria were expanded to include all high-risk infants in the NICU who were referred for therapy (occupational therapy, physical therapy or speech-language pathology) at NICU discharge.

### Study site

Eligible infants were recruited from consecutive discharges from the St. Louis Children’s Hospital Level IV NICU, a 125-bed unit serving urban St. Louis and surrounding areas, during the study time periods (January 2016 to January 2018).

### Baby Bridge program

The Baby Bridge program was developed to minimize the gaps in therapy services that high-risk premature infants often experience after discharge from the NICU. The Baby Bridge program was developed as a partnership between St. Louis Children’s Hospital and the Program in Occupational Therapy at Washington University in St. Louis, in close collaboration with Missouri’s First Steps program. Key features of the Baby Bridge program were designed to address barriers, provide skilled care to high-risk infants, and improve the quality and consistency of services and included:

- The development of a relationship between the Baby Bridge therapist and family that began while the infant still resided in the NICU
- Home visits, scheduled within one week of discharge, that continued weekly until other early therapy services commenced
- Baby Bridge therapist with neonatal expertise, defined as at least one year of education and experience in the NICU setting and with high-risk infants
- Baby Bridge therapist with a commitment to the success of the program; therapist willing to not only provide therapy interventions, but drive the administration and communication needed for program success
- Use of standardized, comprehensive assessments to guide targeted interventions
- Collaboration with the NICU team: including nurses, neonatologists, social workers, and therapists before and after discharge home
- Comprehensive therapeutic approach that included education, advocacy, support, fostering an appropriate environment to promote developmental skill acquisition, providing resources, and providing targeted interventions for the infant and family
- Collaboration with the community therapy providers to ensure the referral reached the source, follow-up on planned intake and evaluations, equipping parents with appropriate language to advocate for services, and communicating current function and medical status to the new providers during the transition.

Data were collected on the number of visits, total payments, and total expenses of the Baby Bridge program and were aggregated by month from January 2016-January 2018.

### Cost of Baby Bridge programming

The Baby Bridge program was implemented from January 2016 to January 2018, and all expenses were documented. The total cost of the Baby Bridge program included the cost of the Baby Bridge therapists, based on time allocation proportional to salaries and benefits, as well as cost of mileage to client locations. Few equipment/supplies, totaling less than $200, were purchased for the provision of Baby Bridge services. This included hand sanitizer, copies, and a few feeding bottles. In addition, the cost of graduate student assistance, which related to administrative costs, was included. The cost of the Baby Bridge therapists was determined based on salary data from Washington University Human Resources, corrected for the percentage of effort dedicated to Baby Bridge services. Fringe benefits were added to salary data, and for the study site were approximately 29%. Mileage reimbursement costs were determined by logs and expense reports filled out by the therapist, with the cost of reimbursement being 53 cents per mile.

#### Baby Bridge therapist

Beginning in January 2016, a single occupational therapist dedicated 50% of a full time equivalent (FTE) position to evaluate and provide treatment through the Baby Bridge program. In mid-November of 2016, the original therapist began to phase out but remained with 50% FTE until December 2016. A new Baby Bridge therapist, an occupational therapist, was hired January 2017 and contributed 33% effort from January 2017 to June 2017, as she built a caseload. She increased her time with the program to 95% FTE for July 2017 to December 2017. Due to other projects, she reduced her time to 75% in January 2018 until the completion of the study. An additional physical therapy (PT) provider, who assisted with caseload overflow based on PT needs of clients, began working in the program in May 2017. The PT worked an average of 1 hour per week (3% FTE) for the Baby Bridge program from May 2017 to December 2017.

The Baby Bridge therapists provided direct services, but also engaged in communication with families, communication with the medical team, scheduling, reminder phone calls/texts, identifying eligible infants, generating referrals, and working with discharge coordinators for referrals to be signed. All infants who received therapy services in the NICU and had therapy needs at discharge were referred to the program. This included infants with prematurity, congenital anomalies, prenatal drug exposure and cerebral injury. Therapy services in the home consisted of direct therapy to the infant to improve neurodevelopmental outcome and feeding, parent education and training, in addition to assessment and resources for parent mental health needs. The Baby Bridge therapist aimed to see an average of 4–5 infants in the home each 8-hour day, with each visit lasting an average of one hour.

### Billing/reimbursements

Baby Bridge services were provided through Washington University Occupational Therapy (WUOT), which is affiliated with the study site NICU. WUOT provides occupational therapy services to clients in a variety of treatment settings and across the lifespan. WUOT manages billing through the Washington University Physicians Billing Services, which coordinates processing of bills, insurance claim submission, and collections. Washington University is a non-profit organization. The billing infrastructure already existed for WUOT, and we aimed to assess the cost of Baby Bridge programming as a program nested under this umbrella, recognizing that this study does not include the cost of the billing infrastructure. Standard OT and PT evaluation services were billed at a rate of $160-$168 per hour, with each evaluation lasting approximately one hour. Therapy evaluations were billed based on evaluation complexity or as a comprehensive developmental screening. Standard OT and PT treatments were billed at a rate of $42 per 15 minutes. The typical therapy visit was one hour in length, resulting in billing of $168 for a treatment session. For treatment sessions, treatments were typically billed in units of time, using the current procedural terminology (CPT) codes “therapeutic activities” or “therapeutic exercise”. However, reimbursement was based on payer fee schedules that were set when contracted with the provider, and this varied across insurers and plans. In addition, for infants with Medicaid, charges for OT and PT were made to one of the three contracted managed care organizations providing Medicaid services to all Missouri children. For private insurance, copayments and/or deductibles may have been the responsibility of the client, depending on the requirements of each plan. Copayments typically ranged from $0-$50 per session. Reimbursements varied within and between plans. For Medicaid, most managed care plans did not have an obligation of copayments.

The generated revenue and anticipated revenue from claims sent to Medicaid and private insurance were calculated. The claims, or total amount billed, as well as the reimbursement, or amount received in payment, were determined overall and by insurance type. When actual reimbursement data was missing for a service provided to a particular patient, reimbursements received from that payer for other patients who received the same service were assumed to apply. The missing data were typically the result of slow payment from Medicaid and other payers and were anticipated to ultimately be posted to the patient’s account, thus representing anticipated revenue to the program. However, payments may be prone to denials and appeals that could take time for processing and resolution.

### Cost analysis

A health economist conducted a cost analysis of Baby Bridge services in order to assess the question of long-term sustainability of the program and to help inform potential replication across other sites with a similar payer mix. This was estimated based on anticipated claims and reimbursement of therapy services, given current billing paradigms.

Sustainability of the program at the rate and level of enrollment observed was assessed by calculating the ratio of total program cost to claim income. The need to interpret findings on sustainability within the context of the scale of the program and the payer mix is discussed.

In addition, revenues and costs were estimated based on full capacity, which was defined as 4 visits per day (21 days per month) for a full time therapist. An estimate of monthly reimbursement at full capacity was made by fitting a linear relationship to the monthly data on number of visits and total reimbursement based on billing/reimbursement history.

## Results

See Table 1 for sample demographic data.

**Table 1:** Sample demographics.

	N (%) Total n = 95
Insurance type	
Public	53 (56%)
Private	42 (44%)
Race	
White	30 (32%)
Black	52 (55%)
Asian	2 (2%)
Other/unknown	11 (11%)
Family factors	
Single mother	46 (48%)
Married mother	49 (52%)
Other siblings at home	36 (38%)

Infants received a range of 1–41 Baby Bridge visits, for an average of 6.8 ± 7.5 (median 3, interquartile range 3–7) Baby Bridge visits.

The total cost of programming from January 2016 to January 2018 was $88,617.98.

Program payments (January 2016-January 2018) totaled $41,067.32, but with Medicaid and private insurance reimbursement imputed at the average rates for each service billed, based on other history of payments, the total becomes $65,126.42.

The average payment for a one-hour visit (billed at a rate of $160-$168) by Medicaid was ($95.37 ± $22.25) and by private insurance was ($128.10 ± $25.74). Private insurance payments were significantly higher than Medicaid payments (P<0.0001).

The total loss (January 2016-January 2018) of the entire program was $16,626.56. However, in 2016, the first year of Baby Bridge programming, the program experienced a loss of $26,460.44 during phase-in, with revenue to the program totaling $11,137.56 and expenses totaling $37,598. In 2017, the second year of Baby Bridge Programming, the program experienced a net gain of $2,968.88, with revenues to the program totaling $53,988.86 and expenses totaling $51,019.98. By Spring 2017, 16 months after implementation, the program began to cover its costs. If the program had continued, it is estimated that by January 2020, 3 years after implementation, the program would have earned enough cumulative revenue to offset initial losses, with ongoing annual profit of approximately $16,308.

Based on full capacity (defined as the full time Baby Bridge therapist working 21 days per month, with an average number of 4 visits per day, amounting to 84 visits per month), reimbursement was estimated at $8,126 per month. Expenses were estimated at $6,017 for the full time therapist and $750 for mileage, totaling $6,767. Based on this estimation, a monthly profit of $1,359 would be expected, which amounts to an annual profit of $16,308 for the Baby Bridge program.

**Fig 1:**
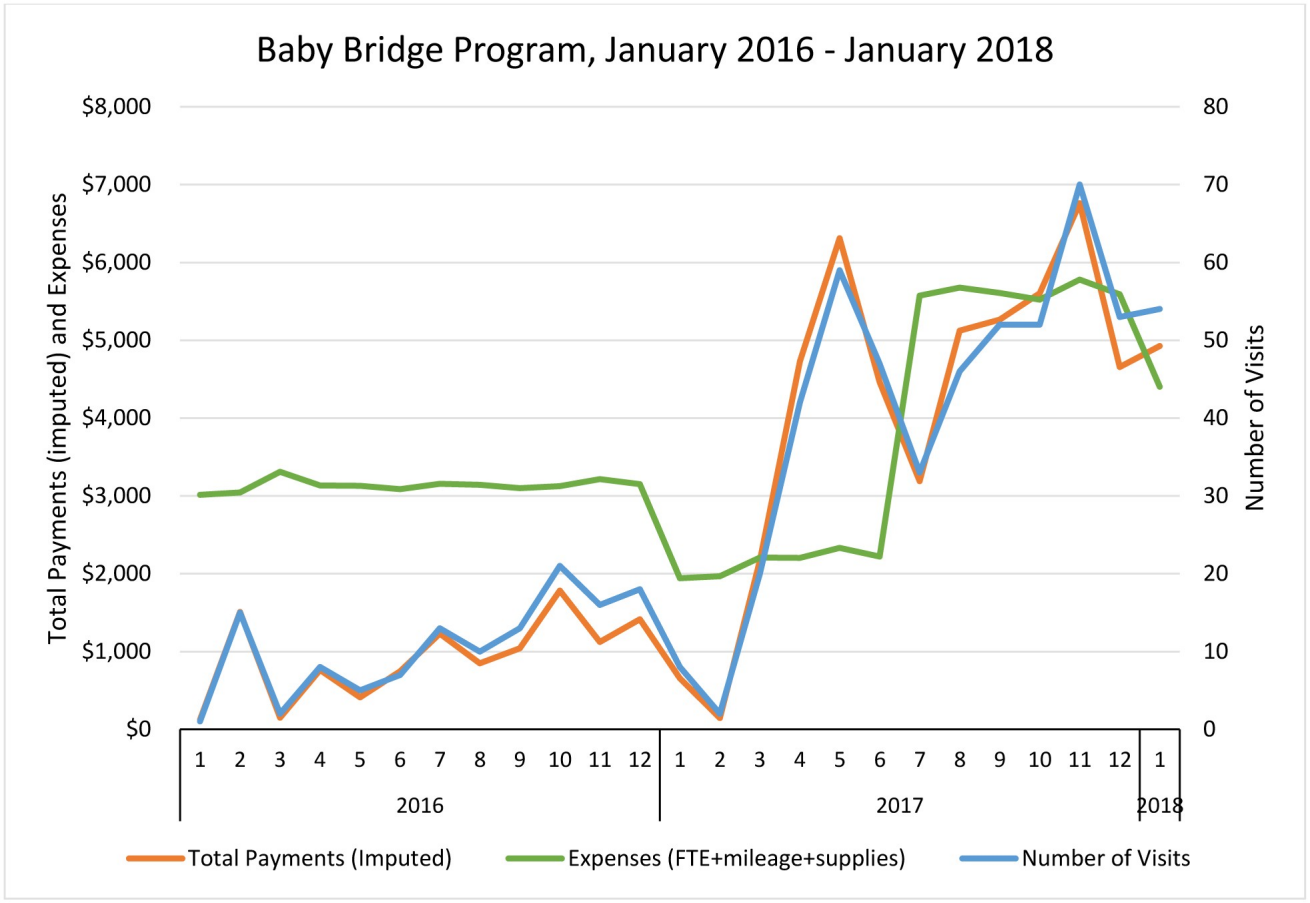
Payments, expenses, and number of Baby Bridge visits from January 2016 to January 2018. The primary Baby Bridge therapist, which has the largest associated costs related to Baby Bridge program expenses, worked 50% of a full time equivalent (FTE) position to evaluate and provide treatment through the Baby Bridge program for the first 11 months. There was a transition in the Baby Bridge therapist one year into programming, with the previous one phasing out and a new therapist phasing in and contributing 33% effort from 1/1/17 to 6/30/17, as she built a caseload. The new Baby Bridge therapist increased her time with the program to 95% FTE from 7/1/17-12/31/17.

Fig 1 summarizes the total payments, expenses, and number of Baby Bridge visits from January 2016 to January 2018.

## Discussion

The key findings of this study are that Baby Bridge programming required an initial commitment to an expected loss; however, after 16 months of operation the program began to cover its costs. It is estimated that after 3 years of implementation, initial start-up costs would have been recuperated given the upward trends in revenues. Following scale-up, with an average of 4 visits per day for a full time Baby Bridge therapist, it is estimated that an annual revenue of $16,308 could be generated by the Baby Bridge program. Organizations can use this information to plan follow-up services aimed at optimizing therapy programming for high-risk infants at NICU discharge, while considering cost versus expense.

Programs aimed at improving access to care require clinical program coordination and/or intake coordinators or navigators, which has been estimated to cost millions of dollars to taxpayers each year through federally mandated therapy programming [35]. Despite this investment in improving access to services, many infants who could benefit from services fail to receive them in a timely manner [19–22]. Those with significant social challenges are at the highest risk of health disparities [28–32]. Previously we have demonstrated a reduction in the time from NICU discharge to receiving therapy in the community, by an average of 85 days, with the Baby Bridge program [33]. Here we demonstrate that the Baby Bridge program can be a sustainable program when nested within a therapy clinic or program with billing infrastructure. Importantly, sustainability is possible even though the Baby Bridge therapist had non-billable administrative time, such as visits to the infant prior to NICU discharge and care coordination with the medical team in the NICU and community-based providers. This is the first report, to our knowledge, that reports cost related to the delivery of early therapy services.

While support of Baby Bridge programming may occur at some sites from philanthropic efforts, understanding the cost and sustainability of such programming is important for replication at other sites. Initial losses were assumed, as it takes time for program development and to build a caseload that fully occupies the therapist’s time; however, the program was sustainable after 16 months of operation. Other hospitals with a similar caseload and payer mix may experience a comparable trajectory when implementing a similar program. Cost of programming is an important factor for others who may consider implementation of Baby Bridge programming; however, cost savings related to health burden is another important area for future inquiry. While this may be challenging to fully ascertain, improving the transition from NICU to home for families may result in improved quality of life, less mental health strain, and decreased readmission rates. Improving access to early therapy services can optimize outcome and reduce the risk of long-term impairment, which can decrease necessary special services later in life and reduce the burden of disability on the infant and family.

Previous research has studied the impact of Baby Bridge programming using a single occupational therapist as the Baby Bridge therapist. However, potential optimization may include expansion of the single provider to a multidisciplinary team equipped to handle the multiple complex challenges of the infant and family following NICU discharge, such as nursing, physical therapy, speech-language pathology, and social work. Data reported here document profits/losses related to a single provider. In addition, revenue/losses here are reliant on the payer mix related to the study site and community served, at which 56% of those served were on Medicaid. This could be different in other settings that provide services to a different payer mix. In addition, reimbursements can be different across different states and insurance providers. Lastly, the cost of therapists specialized in the care of neonates may vary by geographical location.

This study did have limitations. It was conducted in urban St. Louis with a specific payer mix with a high proportion of infants/families with Medicaid. With a different sociodemographic mix and/or in a state with higher Medicaid reimbursement, Baby Bridge programming would likely have higher revenue with quicker achievement of sustainability; therefore, our findings likely represent a lower estimate on timing to recuperate costs and annual revenue. This study relied on billing records that were in various stages of submission and payment, requiring estimation of reimbursement based on previous billing. In some cases, it was unclear why billing had not occurred, so inferences based on billing capacity were also made. Revenue/losses reported here rely on accurate and complete billing/claims and follow-through until payment is received. This study did not account for costs related to billing infrastructure, such as a person to verify payment sources and process claims, address credentialing issues, and deal with denials because it was nested within an already existing clinical program. Replication at sites that do not have a billing infrastructure would need to consider the cost of a billing/administrative person. This study projected profits that extended after the study ended. Fringe benefits at the study site were 29%, which impacts the cost of programming at the study site and may differ at other sites where they may be calculated at a different rate. This study reported on the profit/loss related to Baby Bridge program implementation, but does not report on the social and long-term benefit of such programming. Despite these limitations, this is the first study that we know of that has investigated and reported on sustainability of new programming designed to improve early therapy delivery in order to impact outcomes of high risk infants being discharged from the NICU.

## Supporting information

S1 Data (XLSX)
